# Precision Therapeutics for Monogenic Epilepsies: From Molecular Mechanisms to Disease Modification in Developmental and Epileptic Encephalopathies and Neurodevelopmental Disorders with Epilepsy

**DOI:** 10.3390/jcm15207886

**Published:** 2026-10-13

**Authors:** Debopam Samanta

**Affiliations:** Division of Child Neurology, Department of Pediatrics, University of Arkansas for Medical Sciences, 1 Children’s Way, Little Rock, AR 72202, USA; dsamanta@uams.edu

**Keywords:** functional genomics, genotype–phenotype correlation, personalized medicine, translational neuroscience, molecular diagnostics

## Abstract

**Background**: Developmental and epileptic encephalopathies (DEEs) and neurodevelopmental disorders with epilepsy (NDD-E) are etiologically heterogeneous conditions historically treated with syndrome-based, symptomatic approaches. Advances in genomic sequencing have identified monogenic causes across more than 900 genes, creating opportunities for mechanism-informed and disease-modifying precision therapies. **Methods**: For this narrative review, PubMed/MEDLINE and ClinicalTrials.gov were searched through September 2026 using disorder-, gene-, mechanism-, and therapeutic-modality-specific terms to identify mechanism-informed and disease-modifying therapies for monogenic DEEs and NDD-E. **Results**: Pathogenic mechanisms include gain- or loss-of-function ion-channel abnormalities, altered neurotransmitter receptor/transporter function, impaired synaptic signaling, dysregulated intracellular pathways, metabolic deficiencies, protein misfolding, and transcriptional or epigenetic dysregulation. Across genes such as *SCN1A*, *SCN2A*, *SCN8A*, *KCNQ2*, *KCNT1*, *KCNA2*, *CACNA1A*, *GABA*-receptor subunits, *SLC6A1*, *GRIN* genes, *SYNGAP1*, *STXBP1*, *DNM1*, *SHANK3*, *TSC1/TSC2*, *GNAO1*, *UBE3A*, *MECP2*, and *TCF4*, therapeutic strategies range from repurposed and mechanism-targeted pharmacologic agents (e.g., sodium- and potassium-channel modulators, mTOR inhibitors) to antisense oligonucleotides, RNA interference, gene replacement or augmentation, transcriptional activation, and genome editing. Several programs have advanced from preclinical proof of concept into early- and late-phase human trials, with variant-specific functional classification increasingly guiding therapy selection, particularly distinguishing gain-of-function from loss-of-function disease. **Conclusions**: Precision therapeutics are shifting the treatment paradigm for monogenic DEEs and NDD-E from empiric seizure control toward mechanism-based targeting of dysregulated molecular pathways and gene-based disease-modifying therapies. Realizing this potential will require functional variant classification, delivery technologies capable of durable and cell-appropriate target engagement, and trial designs incorporating developmental and biomarker outcomes beyond seizure frequency alone to demonstrate genuine disease modification.

## 1. Introduction

Developmental and epileptic encephalopathies (DEEs) and neurodevelopmental disorders with epilepsy (NDD-E) comprise an etiologically and mechanistically heterogeneous group of severe neurologic disorders in which epilepsy coexists with developmental impairment, intellectual disability, behavioral and psychiatric manifestations, movement disorders, and other neurologic comorbidities [1,2,3]. In DEEs, developmental dysfunction reflects the combined effects of the underlying neurobiological disorder and the epileptic process, whereas in NDD-E the developmental phenotype is predominantly attributable to the primary etiology, with epilepsy occurring as an associated manifestation [4]. Collectively, these disorders impose a substantial unmet clinical burden, including drug-resistant seizures and status epilepticus, seizure-related injuries, recurrent complications such as aspiration and pneumonia, premature mortality, substantial caregiver burden, and disproportionate healthcare and societal resource utilization [1]. Historically, available treatments have been predominantly symptomatic, relying on empiric or syndrome-directed approaches—for example, seizure control with antiseizure medications (ASMs), and, to a lesser extent, dietary therapies, neuromodulation and epilepsy surgery, alongside symptomatic management of associated neurodevelopmental and systemic manifestations [5,6,7,8,9,10,11,12,13,14,15].

Advances in genomic sequencing have transformed the etiologic and, consequently, therapeutic landscape of these disorders, with monogenic causes now identifiable in a substantial proportion of affected individuals and more than 900 genes implicated across diverse cellular pathways [1,16]. Monogenic disorders provide an especially compelling framework for precision therapeutics because the pathogenic cascade can potentially be traced from a genetic variant to altered protein function, cellular physiology, neural-circuit dysfunction, and ultimately clinical phenotype. These mechanisms encompass gain- or loss-of-function (GoF or LoF) ion-channel abnormalities; altered neurotransmitter receptors and transporters; impaired synaptic vesicle release and postsynaptic signaling; dysregulated intracellular signaling pathways; metabolic deficiencies; abnormal protein folding or trafficking; transcriptional and epigenetic dysregulation [1].

**Figure 1:**
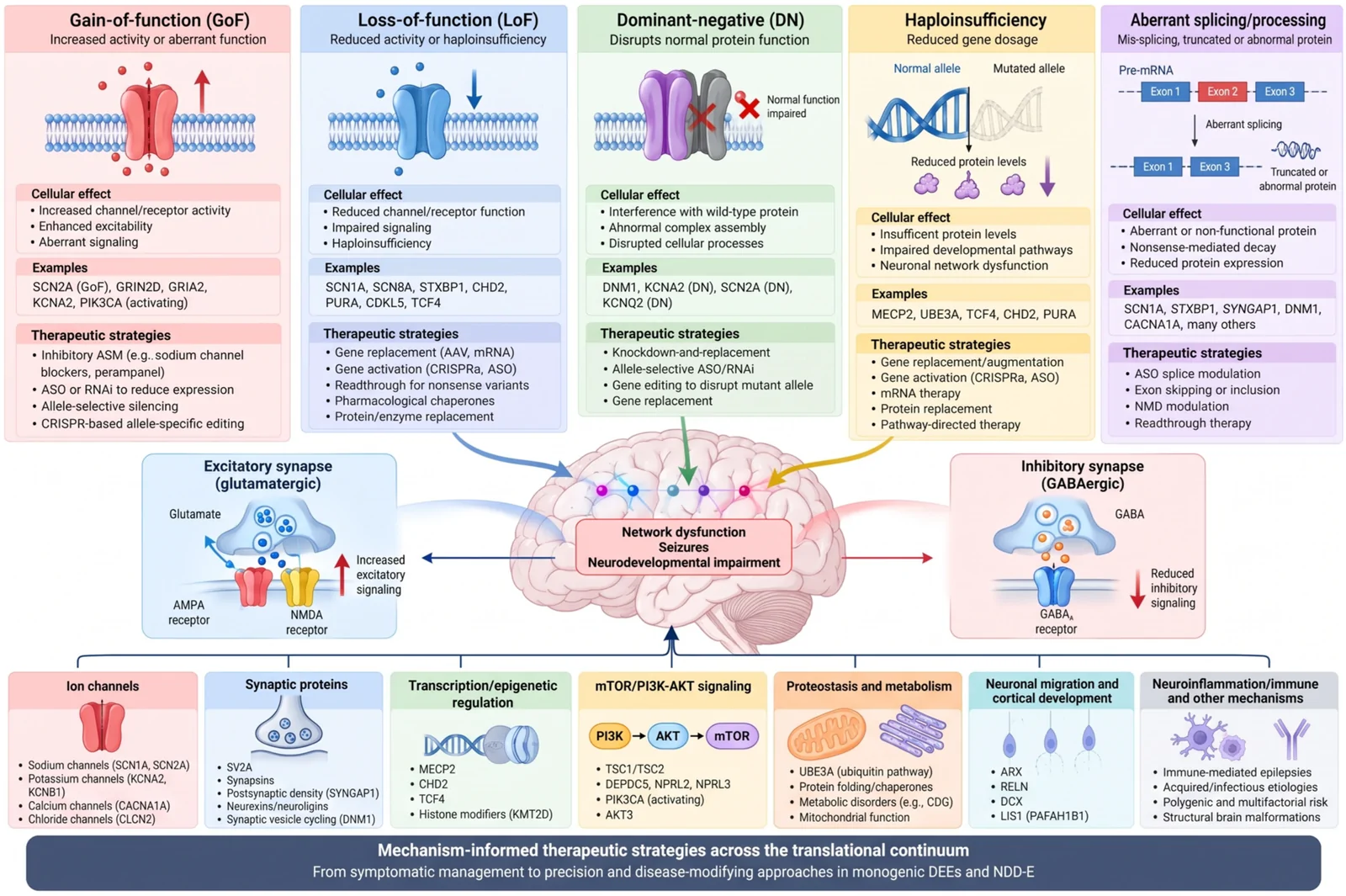
Mechanistic framework for precision and disease-modifying therapies in developmental and epileptic encephalopathies (DEEs). Genetic variants may cause disease through distinct molecular mechanisms, including gain-of-function, loss-of-function, dominant-negative effects, haploinsufficiency, and aberrant RNA splicing or processing, each of which may require a different therapeutic strategy. These molecular defects converge on disruption of excitatory and inhibitory synaptic signaling and broader neuronal networks, resulting in seizures and neurodevelopmental impairment. Additional disease mechanisms involve ion channels, synaptic proteins, transcriptional and epigenetic regulation, mTOR/PI3K–AKT signaling, proteostasis and metabolism, neuronal migration and cortical development, and neuroinflammatory or other acquired processes. Defining the underlying molecular mechanism and functional consequence of a pathogenic variant provides a framework for selecting mechanism-informed therapies across the translational continuum, from symptomatic treatment to precision and potentially disease-modifying approaches. Created in BioRender. Samanta, D. (2026) https://BioRender.com/bwm2ylp.

This mechanistic understanding has created a spectrum of therapeutic opportunities. At one end are mechanism-informed pharmacologic interventions that counteract a defined functional abnormality, such as modulation of ion channels, restoration of deficient metabolites, pharmacologic chaperoning, or inhibition of dysregulated signaling pathways. At the other are potentially disease-modifying molecular therapies—including antisense oligonucleotides (ASOs), RNA modulation, gene replacement or augmentation, transcriptional activation, gene silencing, and genome editing—that seek to correct or compensate for the causal molecular defect itself (Table 1). This review highlights selected examples of monogenic DEEs and NDD-E across diverse functional pathways to illustrate the evolving spectrum of mechanism-informed and potentially disease-modifying therapies (Figure 1).

## 2. Methods

We conducted a narrative review by searching PubMed/MEDLINE and ClinicalTrials.gov through September 2026 for mechanism-informed and potentially disease-modifying therapies for monogenic DEEs and NDD-E. This review provides representative examples spanning major pathogenic mechanisms and therapeutic modalities rather than an exhaustive review of all epilepsy-associated genes. Searches combined disorder-, gene-, mechanism-, and therapeutic-modality-specific terms; detailed search methods, eligibility criteria, evidence categories, and therapeutic classifications are summarized in Table 2. Genes were selected to represent major mechanistic categories and to prioritize disorders for which there is (1) a clinically actionable mechanism-informed therapy, (2) human investigation of a gene- or RNA-directed therapy, or (3) sufficiently advanced preclinical evidence supporting a distinct and potentially translatable precision-therapeutic strategy. The omission of a gene should not be interpreted as indicating lesser biological importance or reduced potential for future precision-therapy development.

## 3. Mechanism-Informed and Disease-Modifying Therapeutic Strategies

### 3.1. Voltage-Gated Ion Channels

#### 3.1.1. *SCN1A*

Most *SCN1A*-related DEE is caused by heterozygous LoF variants producing NaV1.1 haploinsufficiency, preferential impairment of GABAergic interneurons, and the Dravet syndrome phenotype; accordingly, sodium-channel blockers may aggravate seizures and are generally avoided [17]. Conversely, rare gain-of-function *SCN1A* variants may respond to sodium channel blockers. Although valproate, clobazam, stiripentol, fenfluramine, and cannabidiol are effective in reducing seizures in Dravet syndrome, this review focuses specifically on mechanism-informed and disease-modifying therapies [18,19,20,21].

The most advanced RNA therapy is zorevunersen (STK-001), an intrathecal ASO that excludes a naturally occurring nonproductive “poison” exon from *SCN1A* transcripts, thereby increasing productive messenger RNA and NaV1.1 expression from the intact allele [22]. In open-label phase 1–2a and extension studies involving 81 patients aged 2–18 years, initial 70 mg dosing followed by maintenance treatment was associated with median reductions in convulsive-seizure frequency of approximately 59–91% across monthly intervals during the first 20 months, together with exploratory improvements in adaptive behavior [23]. Zorevunersen is now being evaluated in the randomized, double-blind, sham-controlled phase 3 EMPEROR trial in 162 children with Dravet syndrome (NCT06872125). Besides zorevunersen, ION337 is another investigational ASO being evaluated in the phase 1/2 ASCEND study for Dravet syndrome (NCT07531745). Complementary ASO strategies have also shown preclinical efficacy by targeting downstream or parallel regulators. Tau-lowering ASO treatment initiated postnatally reduced epileptiform activity, sudden unexpected death in epilepsy, and autism-like behaviors in *Scn1a* Dravet mice, supporting tau as a disease-modifying network target [24]. Similarly, an RNase-H-activating gapmer ASO targeting *Scn8a* reduced NaV1.6 expression and markedly rescued the Dravet phenotype; a single early dose prolonged survival from approximately three weeks to beyond five months without observed behavioral or electrographic seizures in treated *Scn1a*+/− mice [25]. A third approach targets the *SCN1A* natural antisense transcript (*SCN1A-NAT*) [26]. ASO-mediated inhibition of this regulatory noncoding RNA increased endogenous *SCN1A*/NaV1.1 expression in vitro and in vivo and improved seizures and interneuron excitability in Dravet mice, with target engagement also demonstrated in nonhuman primates [26].

Beyond RNA-based approaches, adeno-associated virus (AAV)-mediated gene regulation provides a complementary strategy to restore NaV1.1 expression by increasing transcription of endogenous *SCN1A*. ETX101 is a one-time intracerebroventricular AAV9 therapy that uses a GABAergic neuron-selective regulatory element and engineered transcription factor to increase endogenous *SCN1A* expression; preliminary open-label POLARIS data showed dose-dependent seizure reduction and neurodevelopmental gains (NCT05419492, NCT06112275, and NCT06283212) [27].

Additional disease-modifying approaches remain preclinical. AAV-delivered CRISPR/dCas9 transcriptional activation of the intact *Scn1a* allele restored interneuron excitability and attenuated febrile seizures and behavioral abnormalities in mouse models without editing the genomic sequence [28,29]. Because full-length *SCN1A* exceeds the packaging capacity of a single AAV vector, a dual-AAV split-intein system with interneuron-selective enhancers has been developed and produced functional NaV1.1, reduced seizures, and improved survival in two Dravet mouse models, providing proof of concept for direct gene replacement [30].

#### 3.1.2. *SCN2A*

Precision treatment of *SCN2A*-related DEE depends critically on variant function. GoF variants, which commonly increase NaV1.2 activity through altered channel gating or increased persistent sodium current, predominate in neonatal and early-infantile epilepsy and often respond preferentially to sodium-channel blockers such as phenytoin and carbamazepine; in contrast, LoF variants are more frequently associated with later-onset epilepsy, intellectual disability, or autism and may respond poorly or worsen with sodium-channel blockade [31,32]. Relutrigine (PRAX-562) represents a potentially more targeted pharmacologic approach for GoF disease because it preferentially inhibits persistent sodium current and exhibits enhanced use-dependent blockade, thereby preferentially suppressing pathologic hyperexcitability while relatively sparing peak sodium current required for physiological neuronal firing [33]. Preclinical studies demonstrated a wider therapeutic index than carbamazepine or lamotrigine [34]. Early clinical findings from the randomized phase 2 EMBOLD study in *SCN2A*- and *SCN8A*-related DEEs, reported in conference abstracts and presentations, showed a 46% placebo-adjusted reduction in motor seizures during the double-blind period, with 33% of participants seizure-free at 16 weeks; sustained seizure reduction was subsequently reported during open-label extension [35,36].

Preclinically, non-allele-selective ASO-mediated reduction in *Scn2a* expression decreased seizure burden and prolonged survival in a GoF mouse model, providing proof of concept for transcript-lowering therapy and supporting its subsequent translation into clinical development [37]. Elsunersen (PRAX-222) is an intrathecally administered ASO designed to reduce *SCN2A* transcript and NaV1.2 expression. In the nine-participant, sham-controlled phase 1/2 EMBRAVE study, company-reported results showed a 77% placebo-adjusted reduction in monthly seizures [38,39]. In a preterm infant with a functionally confirmed GoF variant and prolonged refractory status epilepticus, repeated intrathecal elsunersen treatment was associated with termination of status epilepticus and a >60% reduction in seizure frequency [40].

More recently, individualized allele-selective ASOs were developed for two patients with *SCN2A*-related DEE. These ASOs exploited heterozygous intronic single-nucleotide polymorphisms phased with the pathogenic allele, enabling preferential suppression of the mutant transcript while preserving wild-type *SCN2A* expression. Seizure frequency decreased by 26% and 90% in the two patients, respectively, with reported neurodevelopmental gains and reduced concomitant medication use [41]. This strategy provides greater molecular specificity than nonselective suppression but is constrained by the availability of suitable allele-discriminating variants; compatible SNPs were identified in only 16% of a separate *SCN2A*-related disorder cohort [41]. The molecular advantage of selectively suppressing the pathogenic allele while preserving wild-type expression represents an ideal example of precision therapy. However, major translational challenges remain, including the identification of suitable allele-discriminating variants, individualized oligonucleotide development and validation, manufacturing, regulatory pathways, scalability, cost, and equitable access. Compatible SNPs were identified in only a small proportion of the evaluated cohort, illustrating an important biological constraint on the scalability of this approach.

Conversely, LoF disease requires restoration rather than suppression: AAV-delivered CRISPR activation of the intact *Scn2a* allele corrected neuronal and synaptic abnormalities and reduced seizure susceptibility in haploinsufficient mice, with complementary rescue demonstrated in human stem-cell-derived neurons [42].

#### 3.1.3. *SCN8A*

Treatment of *SCN8A*-related DEEs should also be guided by variant function [43,44]. Most early-onset *SCN8A* epilepsies result from GoF variants that increase persistent or resurgent NaV1.6 current and may preferentially respond to sodium-channel blockers, including phenytoin, carbamazepine, oxcarbazepine, lacosamide, lamotrigine, cenobamate, and relutrigine (PRAX-562; NCT05818553) [45]. In contrast, LoF variants are more often associated with intellectual disability, movement disorders, or generalized epilepsy, in which sodium-channel blockers may be relatively contraindicated. More selective pharmacologic strategies have also been pursued. The NaV1.6-selective inhibitor NBI-921352 demonstrated target-specific preclinical activity, but its phase 2 *SCN8A*-DEE trial (NCT04873869) enrolled only eight participants and was terminated without publicly reported efficacy results [46].

Disease-modifying approaches remain preclinical and are principally directed toward GoF disease. Intracerebroventricular administration of a non-allele-selective *Scn8a* ASO reduced transcript expression by approximately 25–50%, delayed seizure onset, and prolonged survival in R1872W mice; subsequent experiments showed that repeated post-onset ASO administration or a single AAV10-delivered short-hairpin RNA treatment provided seizure protection and survival benefits for up to 12 months [25,47,48].

#### 3.1.4. *KCNQ2*

Treatment of *KCNQ2*-related DEEs should also be guided by variant function. Most pathogenic variants reduce Kv7.2-mediated M-current through haploinsufficiency or dominant-negative effects; sodium-channel blockers—particularly carbamazepine, oxcarbazepine, and phenytoin—frequently control the associated neonatal-onset seizures, although seizure control does not necessarily prevent later neurodevelopmental impairment [49]. Kv7-channel activation provides a more direct mechanism-based approach for LoF variants. Ezogabine (retigabine), a Kv7.2–Kv7.5 channel opener, has shown potential seizure and developmental benefits in small retrospective series, including at least 50% seizure reduction in all five patients with daily seizures in one eight-patient cohort; however, it is no longer commercially available because of safety concerns (retinal pigmentary abnormalities with potential vision loss, blue-gray skin and mucosal discoloration, and urinary retention) and commercial discontinuation [50,51]. Its pediatric granular formulation, XEN496, entered the phase 3 EPIK trial (NCT04639310) but the study was terminated after enrolling only eight participants, precluding definitive efficacy assessment [52]. Gabapentin also enhances Kv7 currents and has produced clinical and electrographic improvement in several patients with functionally confirmed LoF variants, although the evidence remains limited to very small, uncontrolled studies [53,54]. Newer Kv7 openers, including azetukalner and BHVN-7000, are in clinical development for broader epilepsies but have not yet demonstrated efficacy specifically in *KCNQ2*-related disorders [55]. In contrast, for *KCNQ2/KCNQ3* GoF variants, amitriptyline has emerged as a mechanism-informed repurposing strategy through inhibition of excessive Kv7.2/Kv7.3 currents. In vitro rescue and developmental improvement in an initial patient with *KCNQ2* G239S were followed by a 13-patient retrospective cohort reporting improvements across several developmental domains, although persistent benefits after discontinuation limited causal inference [56,57].

Gene-directed therapy remains preclinical. One strategy is indirect compensation: SCN8A-lowering ASO reduced seizures and prolonged survival in a *Kcnq2* LoF mouse model by decreasing excitatory sodium-channel activity [58]. A more directly disease-modifying strategy may involve an allele-selective RNase H1–recruiting ASO that selectively reduces the mutant KCNQ2 transcript while preserving expression from the normal allele [59]. This approach may be particularly relevant for KCNQ2-DEE missense variants with loss-of-function and dominant-negative effects, in which mutant Kv7.2 subunits incorporate into tetrameric channels and impair overall channel function.

#### 3.1.5. *KCNT1*

Most epilepsy-associated *KCNT1* variants produce GoF of the sodium-activated potassium channel KNa1.1 (SLACK), paradoxically promoting network hyperexcitability; therefore, current precision strategies seek to inhibit the channel or reduce its expression [60]. Quinidine inhibits KNa1.1 and has produced occasional substantial responses, but a systematic review found ≥50% seizure reduction in only 20 of 80 patients, with inconsistent responses even among individuals sharing the same variant and frequent dose-limiting QT prolongation [61]. A small randomized crossover trial also found no significant benefit in sleep-related hypermotor epilepsy and identified cardiac toxicity at relatively low serum concentrations [62]. Fluoxetine also directly inhibits KNa1.1 currents. In a prospective open-label study of three patients with KCNT1 whose variants showed in vitro fluoxetine sensitivity, treatment reduced seizure frequency by 25–75%, with reported improvements in visual attention, participation, and muscle tone [63]. More selective therapies have now entered clinical development. ABS-1230 is an orally administered selective KCNT1 inhibitor that inhibited multiple pathogenic variants preclinically and entered a phase 1a study in healthy volunteers, with subsequent evaluation in individuals with *KCNT1*-related epilepsy planned (NCT07156201; NCT07600736) [64].

Preclinical gene-silencing studies provide strong mechanistic support. A gapmer ASO administered after seizure onset produced marked *Kcnt1* knockdown, nearly abolished seizures, prolonged survival, and improved behavior in P905L mice, with even >90% knockdown being tolerated in wild-type animals [65]. This strategy has subsequently advanced to first-in-human treatment. Nakayama et al. treated two two-year-old girls with severe *KCNT1* p.R474H-related epileptic encephalopathy using an intrathecally administered, non-allele-selective *KCNT1*-targeting ASO [66]. Treatment was associated with substantial reductions in seizure frequency and intensity in both patients, providing initial clinical evidence that suppression of *KCNT1* expression can ameliorate the epilepsy phenotype. However, both patients developed ventricular enlargement or hydrocephalus during treatment, highlighting a potentially important toxicity of intrathecal ASO therapy and the need for careful safety monitoring and optimization of dosing and chemistry. A similar non-allele-selective *KCNT1* knockdown strategy is being evaluated with S230815 in the phase 1b/2 KANDLE study (NCT07227857), a first-in-human, open-label, multiple-ascending-dose trial in children with *KCNT1*-related DEE. Additionally, ATL-201, a divalent small-interfering RNA targeting both human and murine *KCNT1*, produced dose-dependent, near-complete seizure suppression lasting at least four months and normalized nest-building behavior in Y777H mice [67].

#### 3.1.6. *KCNA2*

Precision treatment of *KCNA2*-related disorders requires functional classification, as pathogenic variants may produce distinct Kv1.2 channel dysfunction with different therapeutic implications. Potassium-channel inhibition may benefit hyperactive channel variants but could aggravate variants with reduced channel function [68]. The Kv1-channel blocker 4-aminopyridine (fampridine/dalfampridine) can counteract GoF by reducing excessive potassium current. In an international series of individualized trials, 9 of 11 patients with functionally confirmed GoF variants benefited [69]. All six patients with daily absence, myoclonic, or atonic seizures became seizure-free apart from occasional provoked events, while generalized tonic–clonic seizures responded inconsistently. Improvements in ataxia, gait, alertness, speech, and cognition were also reported [69]. In contrast, the Kv1.1/Kv1.2 channel opener pisiferic acid restored channel activity in all 13 LoF and one mixed GoF/LoF Kv1.2 variants tested in vitro, identifying it as a potential therapeutic lead for *KCNA2* LoF variants [70].

Disease-modifying strategies remain preclinical. For the dominant-negative LoF p.P407R variant, a mutation-selective gapmer ASO preferentially suppressed the mutant transcript and restored potassium currents, action-potential duration, and early afterdepolarization abnormalities in cultured rat and human stem-cell-derived neurons, providing proof of principle for allele-selective silencing [71].

#### 3.1.7. *CACNA1A* and *CACNA1E*

CACNA1A encodes the presynaptic P/Q-type CaV2.1 calcium channel, and therapeutic strategies may differ according to the functional effect of the variant [72]. Acetazolamide and calcium-channel blockers such as flunarizine and verapamil have been used as mechanism-informed therapies, particularly for GoF phenotypes, whereas 4-aminopyridine may partially normalize the neuronal dysfunction associated with LoF/haploinsufficiency (by blocking voltage-gated K^+^ channels, prolonging action potentials and thereby enhancing residual CaV2.1-dependent neurotransmitter release) [73,74]. N-acetyl-L-leucine (NALL) has emerged as a complementary downstream strategy, particularly for prominent or progressive cerebellar dysfunction. Rather than directly modulating CaV2.1, NALL appears to enhance cerebellar network compensation and may improve mitochondrial bioenergetics, lysosomal/autophagic function, and neuronal resilience. Early clinical evidence includes a four-patient pediatric series reporting improvements in gait, balance, fine motor function, and speech [75]. A pivotal randomized, double-blind, placebo-controlled crossover phase 3 study is now evaluating NALL in patients aged ≥4 years with genetically confirmed CACNA1A-related disorders (NCT07221292). For *CACNA1E*, GoF of the CaV2.3 R-type calcium channel is the predominant disease mechanism, providing a rationale for R-type calcium-channel inhibition with topiramate; five patients achieved seizure freedom in the original cohort [76]. Gene-directed restoration of expression of these genes remains preclinical, with no gene- or RNA-directed therapy yet in clinical evaluation [77].

### 3.2. Ligand-Gated Receptors and Neurotransmitter Transport

#### 3.2.1. *GABRA1*, *GABRA2*, *GABRB1*, *GABRB2*, *GABRB3*, and *GABRG2*

These genes encode α1, α2, β1, β2, β3, and γ2 subunits of pentameric GABA A receptors, respectively. Pathogenic variants can disrupt receptor expression, folding, trafficking, GABA sensitivity, channel gating, or desensitization, and both LoF and GoF mechanisms are increasingly recognized [78,79,80].

GABA-enhancing therapies, including benzodiazepines, phenobarbital, neurosteroids, and vigabatrin, may compensate for LoF but can worsen excessive GABAergic signaling or cause substantial adverse effects in GoF disease [80,81]. Vinpocetine, identified through functional drug screening as a positive GABA A -receptor modulator, has reduced epileptiform activity or seizures and improved behavioral or cognitive outcomes in individual patients with LoF *GABRB3*, *GABRA1*, and *GABRG2* variants [82,83,84].

Beyond direct receptor modulation, correction of receptor misfolding and impaired membrane trafficking represents another mechanism-informed therapeutic strategy. Positive allosteric modulators such as hispidulin and TP003 can act as pharmacological chaperones, improving folding, surface expression, and function of several LoF α1-containing GABAA receptors in cultured cells and human induced pluripotent stem cells (hiPSCs)-derived neurons [85]. The chemical chaperone 4-phenylbutyrate (4-PBA; clinically available as sodium phenylbutyrate or through the slower-release prodrug glycerol phenylbutyrate) has similarly been investigated as a proteostasis-restoring strategy for trafficking-deficient GABAA receptors, including *GABRA1* and *GABRG2* variants [86]. In *Gabrg2* Q390X models, 4-PBA reduced mutant γ2-subunit accumulation and endoplasmic-reticulum stress, improved wild-type receptor trafficking and GABA-evoked currents, and reduced seizures. These approaches remain preclinical or limited to individualized clinical experience, and no GABAA -receptor gene-specific therapy has yet entered a controlled clinical trial.

#### 3.2.2. *SLC6A1*

*SLC6A1* encodes the GABA transporter GAT-1, and pathogenic variants generally cause LoF through haploinsufficiency, protein misfolding, endoplasmic-reticulum retention, or impaired membrane trafficking, resulting in deficient GABA reuptake and disturbed tonic and phasic inhibition [87]. The leading pharmacologic precision strategy is 4-PBA, which improves folding and surface trafficking of residual wild-type and selected mutant GAT-1 proteins. In cell and knock-in mouse models, 4-PBA restored GABA uptake and reduced seizure burden by approximately 76% [88,89]. An open-label early-phase study (NCT04937062) of glycerol phenylbutyrate enrolled children with *SLC6A1*- or *STXBP1*-related disorders; preliminary reports described clinically meaningful seizure and developmental improvements but reported metabolic acidosis requiring intensive care in one participant; these findings require confirmation in controlled studies [90,91].

Gene replacement has progressed from preclinical studies to early clinical evaluation. AAV9-mediated delivery of human *SLC6A1* normalized electroencephalogram (EEG) abnormalities and improved several behavioral phenotypes in heterozygous and homozygous mouse models, with greater efficacy after early treatment; excessive or broadly distributed expression caused toxicity, supporting cell-specific regulation and careful dose selection [92]. An intrathecal, self-complementary AAV9 vector using an inhibitory-neuron-enriched promoter has entered a phase 1/2 trial, with the first participant reportedly dosed in December 2025 (NCT07173153). Additionally, combined wild-type SLC6A1 cDNA augmentation, achieved by co-transfecting additional wild-type GAT-1–encoding plasmid, with pharmacochaperoning using 4-PBA produced additive functional rescue in patient-variant cellular models [93].

#### 3.2.3. *GRIN1*, *GRIN2A*, *GRIN2B*, and *GRIN2D*

These genes encode subunits of heterotetrameric N-methyl-D-aspartate (NMDA) receptors: two obligatory GluN1 subunits encoded by *GRIN1* combine with two GluN2 subunits encoded by *GRIN2A*, *GRIN2B*, or *GRIN2D* [94]. Pathogenic variants may produce GoF, LoF, or mixed effects through altered agonist sensitivity, channel gating, magnesium blockade, trafficking, or surface expression. NMDA-receptor antagonists—including memantine, dextromethorphan, ketamine, or magnesium—may counteract GoF, whereas co-agonists such as L-serine, D-serine, glycine, or D-cycloserine may augment residual receptor activity in LoF disease [95,96]. Clinical evidence is emerging but remains heterogeneous. In a retrospective series of 34 individuals, 14 of 19 with functionally supported GoF variants experienced improvement with memantine, most frequently in behavior (12/17, 71%) and development (6/12, 50%), with seizure reduction in 7 of 18 (39%) [97]. Radiprodil, a selective negative allosteric modulator of GluN2B-containing receptors, is being evaluated in studies for functionally confirmed GoF *GRIN*-related disorders (NCT05818943; NCT07224581). For LoF variants, a 24-participant, open-label phase 2A study of L-serine reported improvements in selected adaptive, motor, and quality-of-life measures, normalization of EEG abnormalities in five participants, and seizure reduction in one; these findings are being evaluated further in the randomized N-of-1 TAP-GRIN study (NCT07377032) [98]. Additionally, positive allosteric NMDA-receptor modulators and co-agonists partially restored function across 21 LoF variants involving *GRIN1*, *GRIN2A*, and *GRIN2B* in heterologous systems, supporting development of subunit- or variant-selective potentiators [99]. No disease-modifying or gene-directed therapeutic strategies have been registered to date.

### 3.3. Synaptic Release, Scaffolding, and Postsynaptic Signaling

#### 3.3.1. *SYNGAP1*

Most pathogenic *SYNGAP1* variants cause haploinsufficiency of SynGAP, a postsynaptic Ras/Rap GTPase-activating protein that restrains Ras–ERK signaling and AMPA-receptor trafficking; reduced SynGAP therefore accelerates excitatory synapse maturation and increases network excitability [100]. The AMPA-receptor antagonist perampanel has improved electrophysiological abnormalities in *Syngap1* mice and reduced seizures in a very small clinical series [101,102]. Statins have been explored as a mechanism-informed approach to attenuate excessive Ras–ERK signaling. Statins reduce synthesis of mevalonate-derived isoprenoids required for Ras prenylation and membrane localization and may therefore dampen downstream Ras–ERK signaling. Clinical evidence remains limited to individual reports: lovastatin was associated with improvement in repetitive, aberrant, and social behaviors in one adult, atorvastatin with reported cognitive and behavioral improvement in one child, and rosuvastatin with reduced seizure burden in another patient [103,104,105]. Because rosuvastatin has limited blood–brain barrier penetration, transferrin receptor 1 (TfR1)-targeted immunoliposomes have been developed experimentally and increased brain delivery approximately 2.5–2.8-fold in preclinical studies [106]. Importantly, recent data demonstrate major Ras-GAP-independent structural functions of SynGAP at the postsynaptic density, suggesting that modulation of Ras–ERK signaling alone is unlikely to fully correct SYNGAP1 pathophysiology [107].

Regarding disease-modifying strategies, ASOs aimed at restoring expression from the intact *SYNGAP1* allele are advancing through several complementary mechanisms. CMP-002/CMP-SYNGAP-01 is an intrathecally administered ASO that targets a *SYNGAP1* regulatory RNA, thereby relieving transcriptional repression and increasing transcription from the functional allele. In patient-derived neurons and humanized *SYNGAP1* haploinsufficient mice, treatment increased SynGAP protein by up to approximately twofold and reversed deficits in cognition, motor function, and hyperactivity; intrathecal administration in cynomolgus monkeys similarly increased SynGAP across disease-relevant brain regions, supporting progression toward first-in-human evaluation [108].

A separate splice-modulation strategy exploits an endogenous alternative 3′ splice site in *SYNGAP1* that generates an unproductive transcript subject to nonsense-mediated decay (NMD). Genetic deletion of this splice site increased SynGAP expression and rescued electrophysiologic abnormalities in *Syngap1* haploinsufficient mice, while splice-switching oligonucleotides redirected splicing toward productive transcripts and increased SynGAP expression in human iPSC-derived neurons [109]. Closely related work showed that PTBP1/2 binding promotes inclusion of this NMD-associated splice event; steric-blocking ASOs that disrupt PTBP binding increased productive *SYNGAP1* splicing and partially restored SynGAP expression in patient-derived haploinsufficient neurons, with proof of target engagement also demonstrated in mouse brain [110]. Knockdown of the natural antisense transcript *SYNGAP1-AS1* has also been proposed as a means of derepressing the intact *SYNGAP1* allele, although this approach remains exploratory [111].

Several additional disease-modifying strategies remain preclinical. Importantly, genetic restoration of SynGAP expression in adult haploinsufficient mice improved memory, seizure susceptibility, and electrophysiological abnormalities, suggesting that the therapeutic window may extend beyond early development [112]. More recently, AAV-mediated delivery of full-length *SYNGAP1* rescued epileptiform and behavioral phenotypes when administered to juvenile mice [113]. CRISPR activation of the intact allele has also increased SynGAP expression, normalized ERK hyperactivation, and improved working-memory deficits in mice, while increasing *SYNGAP1* expression in patient-derived neurons [114]. In a separate approach, hematopoietic stem-cell gene therapy has shown early proof-of-concept behavioral benefit in mice [115].

#### 3.3.2. *STXBP1*

Most pathogenic *STXBP1* variants cause haploinsufficiency or destabilization of Munc18-1—a presynaptic protein essential for SNARE-complex assembly and neurotransmitter-vesicle release [116]. Chemical chaperones may improve folding and trafficking of selected misfolded proteins. Glycerol phenylbutyrate showed preliminary seizure and developmental benefits in an open-label *STXBP1/SLC6A1* study, although controlled evidence is lacking [117,118].

Regarding disease-modifying therapy, CAP-002 is an intravenously administered, brain-penetrant AAV gene-replacement therapy intended to restore neuronal STXBP1 expression [116]. Its phase 1/2a SYNRGY trial treated one child but was terminated after the participant died from cerebral edema; the underlying cause remained unresolved, emphasizing the safety challenges of systemic, blood–brain barrier-penetrant AAV delivery (NCT06983158). Nevertheless, gene replacement remains biologically compelling: neuron-targeted AAV9 constructs expressed STXBP1 across excitatory and inhibitory neurons, rescued behavioral abnormalities in haploinsufficient mice, and achieved broad central nervous system expression in nonhuman primates, while dorsal-root-ganglion detargeting reduced peripheral toxicity [119]. Subsequent studies suggested that transduction of at least approximately 44% of cortical neurons may be required for phenotypic improvement [120].

#### 3.3.3. *DNM1*

*DNM1* encodes dynamin-1, a presynaptic GTPase required for synaptic-vesicle membrane fission and recycling. Most heterozygous DEE-associated missense variants—particularly those within the GTPase and middle domains—impair oligomerization or vesicle endocytosis through a dominant-negative mechanism, whereas truncating or biallelic variants may produce disease through loss of function [121,122].

For dominant-negative disease, simple gene augmentation may be insufficient because the mutant protein remains expressed. In the *Dnm1* “fitful” mouse model, AAV-delivered RNA interference targeting endogenous *Dnm1* reduced seizures, ataxia, neurodegeneration, and premature mortality [123]. A subsequent mutation-independent knockdown-and-replacement vector simultaneously suppressed endogenous mutant and wild-type transcripts while expressing an RNAi-resistant *DNM1* cDNA, markedly improving survival, growth, synaptic transmission, and transcriptomic abnormalities [124].

#### 3.3.4. *SHANK3*

*SHANK3* encodes a postsynaptic-density scaffold that organizes glutamate receptors, cytoskeletal proteins, and signaling complexes at excitatory synapses; haploinsufficiency from pathogenic sequence variants or 22q13 deletions causes Phelan–McDermid syndrome. Mechanism-informed therapies have focused on enhancing synaptic maturation and plasticity: a small placebo-controlled crossover trial of recombinant IGF-1 reported improvements in social and repetitive behaviors, whereas a larger trial of intranasal insulin showed no significant overall developmental benefit [125,126]. NNZ-2591, an orally administered synthetic analogue of the IGF-1 metabolite cyclic glycine–proline, is intended to normalize impaired synaptic signaling and neuroplasticity. In an 18-participant open-label phase 2 trial, it was generally well tolerated and was associated with improvement in 10 of 14 exploratory assessments, including communication, cognition, behavior, sleep, and gastrointestinal symptoms; these uncontrolled findings are now being tested in the randomized, placebo-controlled phase 3 KOALA trial (NCT07281079) [127].

Preclinical restoration of *Shank3* in adult mice reversed selected synaptic and behavioral abnormalities, supporting a therapeutic window beyond early development [128]. Two AAV-mediated SHANK3 gene-augmentation programs have entered clinical development. JAG201 (tovesangene parvec) uses AAV9 to deliver a neuronally expressed engineered miniSHANK3, whereas RB001, developed by Shenzhen Reborngene Therapeutics, delivers a separately optimized SHANK3 minigene by intracerebroventricular AAV administration (NCT06662188; NCT07014020). Additionally, preclinical studies have identified 3′-UTR–targeting ASOs that increase *SHANK3* expression, producing approximately 1.3–1.6-fold increases in SHANK3 protein in patient-derived hiPSCs and differentiated motor neurons, supporting transcript stabilization/upregulation as a potential strategy for *SHANK3* haploinsufficiency [129].

### 3.4. Mechanistic Target of Rapamycin (mTOR) and G-Protein Signaling

#### 3.4.1. *TSC1* or *TSC2*

Pathogenic loss-of-function variants in *TSC1* or *TSC2*, encoding hamartin and tuberin, disrupt the TSC1–TSC2 complex, impair RHEB inhibition, and constitutively activate mTORC1, providing a direct rationale for pathway-targeted therapy [130]. Vigabatrin is the preferred first-line treatment for TSC-associated infantile epileptic spasms syndrome, although its particular efficacy in TSC may reflect correction of downstream GABAergic dysfunction rather than direct mTORC1 inhibition [131]. EEG-guided preventive vigabatrin delayed seizure onset and reduced infantile spasms and drug-resistant epilepsy in EPISTOP study, whereas PREVeNT trial confirmed delayed and reduced spasms but found no improvement in focal-seizure incidence, drug-resistant epilepsy, or cognition at 24 months [132,133].

Everolimus, an mTOR inhibitor, represents the principal clinically available mechanism-directed therapy and is approved as adjunctive treatment for TSC-associated focal-onset seizures in patients aged ≥2 years, as well as for subependymal giant-cell astrocytomas and renal angiomyolipomas [134]. In the phase III EXIST-3 trial, low- and high-exposure everolimus produced ≥50% seizure-response rates of 28% and 40%, respectively, compared with 15% with placebo, confirming exposure-dependent antiseizure efficacy [135]. Another mTOR inhibitor, sirolimus, showed a nonsignificant trend toward seizure reduction in a small randomized trial of children with refractory TSC epilepsy, while observational data suggest that treatment initiated before epilepsy onset may delay seizures and reduce severe epilepsy [136]. However, mTOR inhibitors generally require sustained administration, incompletely reverse established neurodevelopmental pathology, and carry risks including stomatitis, infection, cytopenias, and metabolic abnormalities; consequently, whether very early mTOR inhibition can prevent epileptogenesis or improve neurodevelopment remains an important question. Early mTOR inhibition is now being tested in several studies: the ViRap study (NCT04987463) compares preventive vigabatrin with rapamycin/sirolimus, TSC-STEPS (NCT05104983) evaluates pre-symptomatic sirolimus in seizure-free infants, and the randomized phase IIb PROTECT trial evaluates sirolimus initiated before four months of age and continued through two years of age, with neurodevelopment as the primary outcome [137].

Next-generation mTORC1 inhibition seeks to overcome limitations of rapalogs. Although rapalogs strongly suppress the S6K/S6 arm of mTORC1, phosphorylation of 4E-BP1 can remain relatively resistant, allowing abnormal cap-dependent translation to persist [138]. The bi-steric mTORC1-selective inhibitor RMC-6272 more completely inhibited both S6K/S6 and 4E-BP1 signaling and rescued rapamycin-resistant abnormalities in proliferation, neurite growth, and translation of neurodevelopmental-disorder-associated transcripts in TSC-deficient human neural progenitor cells [139,140]. RMC-5552 similarly suppressed both S6K/S6 and 4E-BP1/eIF4E signaling in TSC-associated lymphangioleiomyomatosis models while largely sparing mTORC2 [141]. AV078, a CNS-penetrant selective mTORC1 inhibitor, has completed phase 1 development (NCT06205381) and is advancing into clinical evaluation for TSC-associated refractory epilepsy (NCT07680322). A complementary approach is broader inhibition of the dysregulated PI3K–AKT–mTOR signaling axis. Paxalisib (SVG103) is a brain-penetrant PI3K/mTOR-pathway inhibitor now being evaluated in a multinational phase 1b/2a trial in 12 adults with drug-resistant epilepsy associated with TSC, FCD-II, or hemimegalencephaly (NCT07287202).

Additional downstream strategies target consequences of TSC pathway dysregulation outside mTORC1 itself. Increased NKCC1 and reduced KCC2 expression in TSC tubers can produce depolarizing GABAergic signaling, providing a rationale for the NKCC1 inhibitor bumetanide; a small open-label TSC study suggested behavioral improvement but no reduction in seizure frequency [142]. Filamin-A dysregulation represents another mTOR-independent target: genetic FLNA normalization and pharmacologic modulation reduced neuronal dysmorphogenesis and seizures in mTORopathy models, and simufilam subsequently produced dose-dependent attenuation of seizure progression in *Tsc1*-deficient mice [143]. Glutamatergic modulation with the GluN2B-selective negative allosteric modulator radiprodil is also being explored in mTOR-related developmental epilepsies [144].

Gene-restoration strategies remain preclinical but are strongly supported by animal data. AAV-mediated *TSC1* augmentation restored hamartin, reduced mTORC1 activation and neuropathology, and markedly prolonged survival in neuronal and stochastic cerebral *Tsc1*-deficient mouse models, including evidence that treatment can reverse abnormalities across multiple neural cell types [145,146,147]. Because full-length *TSC2* exceeds conventional AAV packaging capacity, an AAV9 vector encoding a condensed tuberin retaining the hamartin-binding and GTPase-activating protein domains was developed and extended median survival from 58 to 462 days while reducing brain pathology in *Tsc2*-deficient mice [146].

#### 3.4.2. *GNAO1*

*GNAO1* encodes Gαo, a highly expressed neuronal G-protein α-subunit that couples inhibitory G-protein–coupled receptors to downstream signaling pathways regulating neurotransmitter release and neuronal excitability [148,149]. Zinc has emerged as a variant-informed experimental therapy because it restores GTP hydrolysis and protein interactions for selected mutant Gαo proteins; in a first-in-human Gly203Arg case, oral zinc acetate was associated with cessation of daily dyskinetic crises, improved dystonia scores, and reduced seizures over 11 months [150]. More recently, an open-label pilot study of 13 individuals found oral zinc feasible and generally well tolerated [151]. Exploratory findings suggested possible improvements in gross motor function, daily activities, and disability, but there was no clear improvement in blinded movement-disorder severity or seizure occurrence. High-throughput screening has also identified a small molecule that preferentially inhibits abnormal GTP loading by the recurrent T241_N242insPQ protein, although efficacy currently remains limited to preclinical biochemical models [152]. In *GNAO1*-related DEE, Gαo dysfunction disrupts striatal dopamine–adenosine signaling. By antagonizing adenosine A2A receptors, caffeine may counteract this abnormal signaling. Caffeine improved motor phenotypes in *GNAO1 C. elegans* and G203R mouse models, although it has not been systematically studied in humans [153,154].

Both augmentation and mutant-allele suppression are being pursued as disease-modifying strategies. In preclinical studies, intrastriatal AAV9-mediated wild-type *GNAO1* augmentation improved hyperlocomotion in R209H mice, while intracerebroventricular augmentation corrected behavioral and neuronal abnormalities in C215Y mice and showed broader activity across *GNAO1* variants in *C. elegans* models [155,156]. Allele-selective ASOs suppressed the pathogenic E246K transcript and improved molecular abnormalities in patient-derived cells, while an AAV–RNA-interference construct has been developed to silence the recurrent c.607G>A (p.Gly203Arg) allele [157,158]. Tianasen, an intrathecally administered allele-specific ASO targeting c.607G>A, entered an open-label phase I/II dose-escalation study (NCT07363603), representing the first clinical evaluation of a gene-directed therapy for *GNAO1* DEE.

### 3.5. Epigenetic and Transcriptional Regulation

#### 3.5.1. Ubiquitin-Protein Ligase E3A (*UBE3A*)

Loss of neuronal expression of maternally inherited *UBE3A*, which encodes an E3 ubiquitin ligase, causes Angelman syndrome through maternal deletion, pathogenic sequence variants, imprinting defects, or paternal uniparental disomy [159]. The principal precision strategy exploits the intact but normally silenced paternal allele by suppressing the long noncoding antisense transcript *UBE3A-ATS.* Three intrathecal ASOs have reached clinical development [160]. Rugonersen reduced EEG delta power dose-dependently and produced exploratory developmental gains beyond natural-history expectations in the 61-participant, open-label phase 1 TANGELO study; a randomized phase 3 BEACON trial (NCT07605429) is now underway [161]. Obudanersen (ION582) showed preliminary multidomain improvements in the phase 1/2 HALOS study and is being evaluated in the randomized phase 3 REVEAL trial (NCT06914609). In contrast, apazunersen (GTX-102) did not improve Bayley-4 cognitive scores or the Multidomain Responder Index in the 129-participant phase 3 Aspire trial, despite earlier open-label signals [162].

Direct gene replacement has also entered clinical development. MVX-220 is an AAV-based therapy designed to deliver a functional *UBE3A* copy to neurons through a single intra–cisterna magna injection, with the phase 1/2 ASCEND-AS trial (NCT07181837) currently ongoing. Preclinical approaches seeking durable paternal-allele reactivation include AAV-delivered Cas9, RNA-targeting Cas13, artificial transcriptional repressors, epigenetic editing, and nickase Cas9, several of which restored UBE3A expression and improved selected phenotypes in mice [163,164,165].

#### 3.5.2. Methyl-CpG Binding Protein 2 (*MECP2*)

Loss-of-function variants in X-linked *MECP2*, which encodes a methylated-DNA–binding chromatin regulator, cause Rett syndrome through widespread disruption of activity-dependent transcription, synaptic maturation, and neuronal network homeostasis; clinical severity is influenced by variant type and tissue-specific X-chromosome inactivation [166]. MeCP2 deficiency is associated with abnormalities in BDNF and IGF-1-related pathways, and IGF-1 activates intracellular pathways overlapping with BDNF signaling, particularly PI3K–AKT and MAPK/ERK. Trofinetide, a synthetic analogue of the IGF-1-derived tripeptide glycine–proline–glutamate, modestly improved Rett Syndrome Behavior Questionnaire and Clinical Global Impression–Improvement scores in the phase III LAVENDER trial and is FDA-approved for Rett syndrome in patients aged ≥2 years [167].

Two dosage-regulated AAV9 gene-replacement programs have advanced clinically because both insufficient MeCP2 and excessive expression—as occurs in *MECP2* duplication syndrome—are neurologically harmful. Intrathecal TSHA-102 delivers a miniaturized *MECP2* transgene incorporating a miRNA-responsive autoregulatory element and is being evaluated in the REVEAL phase I/II and pivotal studies (distinct from the obudanersen/ION582 REVEAL study in Angelman syndrome); company-reported interim data from 12 participants indicated that all gained or regained at least one developmental milestone (NCT05606614). Intracerebroventricular NGN-401 delivers full-length *MECP2* using EXACT transgene regulation; company-reported phase I/II data described developmental gains in all 10 evaluable participants at the selected dose, and a registrational study has completed dosing (NCT05898620).

Preclinical alternatives seek to restore endogenous regulation rather than introduce an additional gene copy. CRISPR-based epigenome editing reactivated the silent wild-type *MECP2* allele on the inactive X chromosome and corrected morphological and electrophysiological abnormalities in human Rett neurons, while pharmacologic or miRNA-mediated interference with X-chromosome silencing improved cellular or mouse phenotypes [168,169]. Nuclease-free homologous-recombination editing has corrected pathogenic *MECP2* variants while retaining endogenous regulatory elements, and synthetic miRNA feedback circuits have maintained MeCP2 expression within a safer therapeutic range in AAV-treated mice [170,171].

*MECP2* duplication syndrome (MDS) results from increased dosage and overexpression of the dosage-sensitive transcriptional regulator MeCP2, making normalization of *MECP2* expression a particularly direct precision-therapeutic strategy. ASOs targeting *MECP2* mRNA reduce MeCP2 expression and reversed molecular, electrophysiologic, and behavioral abnormalities even in symptomatic MDS mice, while subsequent studies in a fully humanized MDS model demonstrated dose-dependent normalization of MeCP2 and rescue of behavioral and transcriptional abnormalities, supporting reversibility of established disease [172]. Patient-derived MDS neurons further demonstrated that *MECP2*-targeting ASOs partially normalize dysregulated transcriptional programs and abnormal neuronal morphology [173]. This strategy has now advanced clinically with ION440, an *MECP2*-lowering ASO being evaluated in an early-phase clinical trial (NCT06430385). An alternative preclinical RNA-silencing strategy uses AAV9-delivered high-fidelity Cas13Y to degrade *MECP2* mRNA; this approach normalized downstream gene expression, improved behavioral abnormalities, and prolonged survival in MDS mice, with substantial *MECP2* knockdown also demonstrated in nonhuman primates [174].

#### 3.5.3. *TCF4*

Pitt–Hopkins syndrome (PTHS) is caused predominantly by loss-of-function variants or deletions of *TCF4*, resulting in haploinsufficiency of TCF4, a dosage-sensitive basic helix–loop–helix transcription factor that regulates broad gene networks involved in neurogenesis, neuronal differentiation, synaptic function, excitability, and oligodendrocyte development [175,176]. Experimental studies have identified several potentially targetable downstream consequences of TCF4 deficiency. In a phase 2 open-label PTHS-001 trial, NNZ-2591 was associated with clinician- and caregiver-reported improvements across all PTHS-specific efficacy measures, particularly social interaction, communication, motor skills, and cognition [177]. Loss of TCF4 leads to ectopic *SCN10A*/NaV1.8 expression, and pharmacologic NaV1.8 inhibition has rescued electrophysiologic, respiratory, and behavioral abnormalities in PTHS models [178]. This strategy has recently received preliminary clinical support from a single-patient report in which repurposed nicardipine, which inhibits NaV1.8 in addition to its established calcium-channel activity, was associated with improvements across several developmental domains and restlessness [179]. Other mechanism-informed approaches include increasing TCF4 expression through WNT/β-catenin or epigenetic modulation and histone deacetylase (HDAC) inhibition, with HDAC2 knockdown and broad HDAC inhibition rescuing memory-related phenotypes in Tcf4-haploinsufficient mice [180,181]. This mechanistic rationale has now advanced to clinical evaluation, with an exploratory study of vorinostat (a HDAC inhibitor) assessing safety and efficacy in individuals with PTHS (NCT07150026). TCF4 deficiency also disrupts oligodendrocyte maturation and myelination, and the promyelinating agents clemastine, sobetirome, and the brain-penetrant sobetirome prodrug Sob-AM2 improved myelination, electrophysiologic abnormalities, and behavioral phenotypes in PTHS mice [182,183].

More direct disease-modifying strategies seek to restore TCF4 itself. Genetic reinstatement of *Tcf4* during early postnatal development improved behavioral abnormalities, partially corrected electrophysiologic abnormalities, and normalized expression of TCF4-regulated genes in a mouse model, providing proof of concept that at least some consequences of TCF4 haploinsufficiency remain reversible after birth [184]. These findings have supported the development of direct TCF4 gene replacement; an investigational recombinant AAV9 vector encoding TCF4 (MZ-1866) has received FDA orphan-drug designation for intracerebroventricular injection, representing translation of TCF4-restoration strategies toward clinical development (NCT07135050). Additionally, a phase 1/2 randomized, double-blind, placebo-controlled study was designed to evaluate intravenous AlloRx^®^ mesenchymal stem cells as a potential treatment for Pitt–Hopkins syndrome (NCT05165017).

Selected additional genes are summarized in Table 3 [185,186,187,188,189,190,191,192,193,194,195,196,197,198,199,200,201,202,203,204,205,206,207,208,209].

### 3.6. Metabolic Disorders

Several DEEs and NDD-Es result from disruptions in cellular metabolism, protein synthesis, trafficking, or degradation pathways. Precision strategies for these disorders often target the downstream metabolic or cellular consequences of the underlying genetic defect, including substrate reduction, enzyme or protein replacement, metabolic supplementation, molecular chaperones, and gene-directed therapies that restore deficient protein function or correct abnormal metabolite accumulation (Table 4).

## 4. Limitations

This review has several limitations. First, its narrative design does not include a formal risk-of-bias assessment or quantitative synthesis. Second, there is potential for selection and publication bias, particularly toward positive preclinical findings and company-reported results. Third, many clinical observations are based on small, uncontrolled, open-label studies, case reports, conference abstracts, preprints, or press releases and should therefore be interpreted cautiously. In addition, the clinical-trial landscape is evolving rapidly, and trial status and developmental-stage information are accurate only as of the final search date. The genes and disorders included are illustrative rather than exhaustive.

The review intentionally focuses on monogenic disorders because they provide a particularly direct framework for linking a defined molecular mechanism to a precision-therapeutic strategy. However, DEEs and NDD-E are etiologically heterogeneous and are not restricted to monogenic disorders. Structural brain abnormalities and malformations of cortical development, acquired or infectious insults, immune-mediated mechanisms, polygenic or multifactorial susceptibility, and other genetic or epigenetic mechanisms may also contribute to these phenotypes. Accordingly, the precision-therapeutic framework discussed in this review should not be generalized to all DEEs or NDD-E.

## 5. Future Directions

The next phase of precision therapeutics for monogenic epilepsies will require closer integration of molecular diagnosis, functional variant classification, therapeutic modality, and developmental timing. For gain-of-function and dominant-negative disorders, allele-selective silencing or editing may offer greater specificity than nonselective suppression, whereas haploinsufficiency may be addressed through transcript upregulation, splice modulation, gene augmentation, or transcriptional activation. Across modalities, major translational challenges include brain-wide and cell-type-appropriate delivery, durable but physiologic target engagement, immunogenicity, dose control, and treatment of disorders in which the therapeutic window begins before clinical seizures. Trial design must also evolve beyond seizure frequency alone by incorporating validated developmental, behavioral, electrophysiologic, biomarker, and caregiver-relevant outcomes that are sensitive to meaningful change in rare and heterogeneous populations. Natural-history cohorts, quantitative biomarkers, and trial-ready endpoints will be essential for distinguishing true disease modification from symptomatic improvement.

## 6. Clinical Implications

As precision therapies for DEEs and NDD-E continue to evolve, their translation into routine clinical practice will require earlier recognition and molecular diagnosis. Children with epilepsy accompanied by developmental delay, regression, or other neurodevelopmental features should prompt timely neurologic and genetic evaluation using contemporary genetic testing approaches. Importantly, treatment decisions should increasingly consider the functional consequence of the specific variant rather than the gene diagnosis alone, as this may guide therapy selection, identify potentially aggravating treatments, and inform eligibility for precision-therapy trials. Molecular diagnosis can also improve genetic and prognostic counseling and facilitate earlier access to emerging therapies. As these approaches enter clinical practice, clinicians will need to clearly distinguish established treatments from investigational therapies and counsel families regarding the remaining uncertainty surrounding their long-term developmental and disease-modifying benefits.

## 7. Conclusions

Precision therapeutics are changing the treatment framework for monogenic DEEs and NDD-E from syndrome-based seizure suppression toward interventions matched to causal molecular mechanisms. Functional characterization is increasingly central: the same gene may require channel inhibition, channel activation, transcript reduction, allele-selective silencing, or restoration of gene expression depending on the pathogenic mechanism. Several RNA- and gene-directed programs have now progressed from preclinical proof of concept to human trials, while pathway-directed pharmacologic strategies provide nearer-term opportunities for selected disorders. The principal challenge is no longer simply identifying a genetic diagnosis, but translating that diagnosis into a therapeutically actionable mechanism and demonstrating clinically meaningful benefit across seizures, development, behavior, and neurologic function. Continued integration of genomics, functional biology, delivery technologies, natural-history data, and rigorous outcome measures will determine whether these approaches achieve durable disease modification.

## Figures and Tables

**Table 1:** Disease-modifying molecular therapeutic strategies relevant to monogenic developmental and epileptic encephalopathies and neurodevelopmental disorders with epilepsy.

Therapeutic Strategy	Principal Mechanism	Potential DEE/NDD-E Candidates or Examples	Major Advantages	Major Limitations/Challenges	Current Translational Stage
Protein replacement	Administration of the deficient functional protein; cell-penetrating or secretable proteins may permit uptake by neighboring cells and partial cross-correction.	*CDKL5* deficiency (TAT-CDKL5); selected enzyme/protein deficiencies	Does not alter DNA; potentially titratable and reversible; avoids vector-genome persistence	Short protein half-life; repeated administration; BBB penetration and intracellular delivery; immunogenicity; inadequate brain distribution	Preclinical
Enzyme replacement/cross-correction	Provides functional enzyme that can be taken up by deficient cells, particularly when secreted proteins undergo receptor-mediated uptake.	Selected lysosomal/metabolic NDD-E; potentially secreted or engineered proteins	Established therapeutic concept in metabolic diseases; adjustable/discontinuable dosing	Limited applicability to nonsecreted neuronal proteins; CNS delivery; repeated dosing	Established clinical use in selected metabolic disorders; predominantly preclinical in DEE/NDD-E
Gene replacement/gene augmentation	Functional cDNA is delivered, usually by AAV, to compensate for insufficient endogenous gene function; the pathogenic allele remains unchanged.	*SLC6A1*, *STXBP1*, *CDKL5*, *SHANK3*, *TSC1*; mini-*TSC2*; potentially *SYNGAP1*	Potential single administration; durable neuronal expression; mutation-independent within a gene	AAV cargo limit; dosage control; overexpression toxicity; targeting; anti-AAV immunity; irreversible exposure; developmental timing	Preclinical–early clinical
Dual-AAV/split-intein gene replacement	Large genes are divided between vectors and reconstituted at the RNA or protein level after cotransduction.	Large genes exceeding single-AAV capacity, e.g., *SCN1A*	Extends gene replacement to genes too large for conventional AAV	Requires efficient cotransduction; reduced effective dose; complex manufacturing; incomplete/aberrant reconstitution	Preclinical
Knockdown-and-replacement	Suppresses endogenous mutant and wild-type transcripts while expressing an RNAi-resistant functional cDNA.	Dominant-negative disease, e.g., *DNM1*; conceptually *KCNQ2*, *KCNA2*	Addresses dominant-negative toxicity that simple augmentation cannot correct; potentially mutation-independent	Requires balanced knockdown/replacement; complex construct; incomplete suppression or excessive replacement	Preclinical
CRISPR/Cas nuclease editing	Guide RNA directs Cas nuclease to DNA, generating a double-strand break followed by NHEJ-mediated disruption or, less efficiently, HDR-mediated correction.	Dominant toxic/GoF alleles; *UBE3A-ATS*; potentially selected monogenic epilepsies	Permanent intervention; can directly eliminate a pathogenic allele or regulatory sequence	Genotoxicity; off-target editing; chromosomal rearrangements; inefficient HDR in postmitotic neurons; immunogenicity/delivery	Preclinical
Allele-selective genome editing	Variant- or haplotype-specific CRISPR selectively disrupts or corrects the pathogenic allele while preserving the normal allele.	*SCN2A*, *SCN8A*, *KCNT1*, *KCNQ2*, *KCNA2*, *GNAO1*	Preserves normal allele; potentially permanent	Highly variant-specific; PAM/sequence constraints; individualized development; off-target allele editing	Preclinical/conceptual
Base editing	Cas nickase–deaminase directly converts selected bases without double-strand DNA cleavage.	Editable point mutations; potentially *SCN1A*, *SCN2A*, *MECP2*, *CDKL5*, and *GRIN* disorders	Permanent single-base correction; avoids double-strand breaks; suitable for postmitotic neurons	Restricted nucleotide changes; bystander editing; sequence/PAM constraints; large cargo; off-target DNA/RNA editing	Preclinical
Prime editing	Cas nickase–reverse transcriptase and prime-editing guide RNA write specified substitutions or small insertions/deletions into genomic DNA.	Broad range of point mutations and small indels across monogenic DEEs	Greater sequence flexibility than base editing; potentially precise and permanent	Large machinery; difficult CNS delivery; variable in vivo efficiency; pegRNA design; uncertain long-term safety	Preclinical/conceptual
CRISPR activation (CRISPRa)	dCas9 recruits transcriptional activators to endogenous promoters/enhancers to increase transcription without changing DNA sequence.	*SCN1A*, *SCN2A*, *SYNGAP1* and other dosage-sensitive genes	Preserves endogenous structure, isoforms, and regulation; bypasses cDNA size limits; avoids DNA breaks	Large payload; often dual vectors; sustained Cas expression; risk of excessive activation	Preclinical
CRISPR interference (CRISPRi)	dCas9 fused to transcriptional repressors decreases endogenous transcription without DNA cleavage.	GoF/toxic overexpression; potentially *SCN2A*, *SCN8A*, *KCNT1*	Tunable repression; no double-strand breaks; can target transcription rather than individual variants	Sustained expression; incomplete/excessive suppression; large delivery system	Preclinical
Epigenome editing	dCas9–chromatin modifier alters DNA methylation or histone state to activate or repress endogenous genes.	*UBE3A*; potentially *MECP2* and other transcriptional/dosage disorders	May reactivate endogenous alleles without DNA sequence alteration; potentially prolonged effect	Off-target/spreading effects; uncertain durability/reversibility; complex CNS delivery	Preclinical
mRNA replacement	Synthetic modified mRNA provides a transient template for translation of the missing protein.	*CDKL5*, *STXBP1*, *SLC6A1*, *TSC1/2*; metabolic genes	No permanent DNA change; transient and dose-adjustable; accommodates relatively large coding sequences	RNA instability; innate immunity; repeated dosing; CNS distribution and endosomal escape	Preclinical
ASO: RNase-H1 gapmer	DNA-containing ASO hybridizes to target RNA and recruits RNase H1, causing transcript degradation.	GoF/toxic transcripts: *SCN2A*, *SCN8A*, *KCNT1*; allele-selective *GNAO1/KCNA2/KCNQ2* strategies	Sequence-specific; adjustable dosing; established CNS oligonucleotide chemistry	Repeated intrathecal dosing; excessive knockdown may cause LoF toxicity; inflammatory/CSF effects; variable distribution	Preclinical–early clinical
Allele-selective ASO	ASO recognizes the pathogenic variant or a linked heterozygous polymorphism and preferentially degrades the mutant transcript.	*SCN2A*, *KCNA2*, *KCNQ2*, *GNAO1*	Preserves normal-allele expression; attractive when global suppression is undesirable	Often personalized; exploitable sequence differences not universal; manufacturing complexity; incomplete discrimination	Case-level clinical evidence; otherwise preclinical
ASO: splice switching/steric blocking	Fully modified ASO masks splice sites or regulatory elements without RNA degradation to alter exon inclusion/exclusion or restore productive splicing.	*SCN1A* poison-exon exclusion; *SYNGAP1* unproductive splice-site suppression; variant-specific splice defects	Can increase protein; preserves endogenous regulation; no permanent genomic editing	Requires exploitable splice biology; repeated dosing; transcript/cell-type variability; potential incorrect splicing	Clinical development
Targeted augmentation of nuclear gene output (TANGO)	Splice-switching ASO suppresses naturally occurring nonproductive/poison exons that trigger NMD, increasing productive transcript and protein from the intact allele.	*SCN1A*—zorevunersen; conceptually other haploinsufficient genes	Mutation-independent for many LoF variants; amplifies endogenous functional allele; maintains normal protein sequence	Restricted to genes with exploitable nonproductive splicing; endogenous transcription imposes ceiling; repeated intrathecal dosing	Late clinical development
ASO targeting regulatory RNA/transcriptional “dimmer switches”	Steric-blocking ASO targets regulatory RNA to remove repression of the corresponding coding gene and increase endogenous expression.	*SYNGAP1*—CMP-002/CMP-SYNGAP-01; potentially other haploinsufficient genes	Increases endogenous full-length protein; preserves native regulation; potentially mutation-independent	Regulatory RNAs incompletely characterized; potential off-target transcriptional effects; repeated dosing; few validated targets	Early clinical development
Natural antisense transcript (NAT) suppression	ASO, siRNA, or editing suppresses an antisense RNA that normally silences or inhibits the functional allele.	*UBE3A-ATS*; *SCN1A-NAT*; exploratory *SYNGAP1-AS1*	Reactivates endogenous gene; may retain normal isoform structure	Complex target biology; excessive expression; repeated ASO treatment; some approaches remain preclinical	Clinical development–preclinical, depending on target
ASO masking 3′-UTR regulatory elements	Steric-blocking ASO masks destabilizing miRNA/RNA-binding-protein sites, increasing transcript stability or translation.	Preclinical *SHANK3* upregulation	Increases endogenous protein without manipulating DNA or splicing; comparatively simple design	Regulatory-site uncertainty; moderate expression gain; repeated dosing; off-target RNA-network effects	Preclinical
siRNA-mediated RNA interference	Double-stranded siRNA enters RISC; the guide strand directs sequence-specific target-mRNA cleavage.	*KCNT1*; experimentally *SCN8A* and other overactive genes	Potent catalytic silencing; relatively straightforward sequence design; potentially prolonged effect	CNS distribution/endosomal escape; repeated dosing unless vectorized; seed effects; excessive suppression	Preclinical
Vectorized shRNA/miRNA-based silencing	AAV expresses shRNA or engineered miRNA processed into an RNAi guide, producing sustained RISC-mediated knockdown.	*SCN8A*; *DNM1* knockdown-and-replacement	Potential single administration; durable silencing; cell-specific promoters	Difficult to reverse; saturation of endogenous RNAi machinery; durable off-target effects; dose control	Preclinical
miRNA replacement/inhibition	miRNA mimics restore deficient regulatory miRNAs; antimiRs inhibit pathogenic miRNAs and derepress target transcripts.	Downstream pathway modulation in synaptic, mTOR, inflammatory, and ion-channel DEEs	Can coordinately modify pathological networks	Lower target specificity; broad transcript effects; unpredictable network effects; delivery challenges	Preclinical
RNA editing—ADAR recruitment	Guide RNA recruits endogenous/exogenous ADAR to convert adenosine to inosine, read as guanosine during translation.	A→G-equivalent corrections; potentially selected *MECP2*, channel, and receptor variants	RNA correction without permanent DNA alteration; reversible; may exploit endogenous machinery	Mainly A→I(G) editing; repeated/persistent guide delivery; bystander/off-target editing; CNS delivery	Preclinical
RNA trans-splicing	Engineered RNA replaces part of an endogenous transcript through spliceosome-mediated trans-splicing to generate corrected full-length mRNA.	Large genes or clustered pathogenic variants	May correct multiple variants in a region; avoids permanent DNA editing; may bypass full-gene viral cargo limits	Low/variable efficiency; competition with cis-splicing; aberrant trans-splicing	Preclinical/conceptual
Suppressor tRNA therapy	Engineered tRNAs recognize premature termination codons and insert an amino acid, permitting translation to continue.	Nonsense variants in *SCN1A*, *STXBP1*, *CDKL5*, *SYNGAP1* and others	Potentially gene-agnostic for shared stop codons; restores full-length protein without DNA editing	Inserted amino acid may differ from native residue; normal-stop readthrough; delivery/expression control	Preclinical
Small-molecule nonsense suppression/translational readthrough	Compounds such as ataluren promote near-cognate tRNA incorporation at premature termination codons.	Nonsense variants; clinically tested in *SCN1A* Dravet syndrome and *CDKL5* deficiency	Oral, mutation-class approach; avoids gene/vector delivery	Sequence/context dependent; requires residual transcript; CNS exposure; negative/limited findings in the examples discussed	Early clinical evidence
Nonsense-mediated decay modulation	Inhibits degradation of premature-stop-containing transcripts to increase substrate for readthrough or other rescue approaches.	Selected nonsense variants across LoF DEEs	May synergize with readthrough/RNA editing; increases mutant-transcript availability	NMD is fundamental quality control; broad inhibition may stabilize abnormal transcripts and cause toxicity	Preclinical/conceptual
Small-molecule pharmacological chaperoning/proteostasis rescue	Stabilizes misfolded proteins, improves ER processing/membrane trafficking, or increases residual protein abundance.	*SLC6A1* and GABA\(_A\)-receptor genes; other trafficking-defective channels/receptors	Oral/systemic therapy potentially feasible; variant-informed; can rescue residual protein	Limited to variants retaining rescuable function; off-target proteostasis effects; incomplete rescue; chronic therapy	Preclinical–early clinical
Pathway-directed molecular therapy	Targets a causal downstream signaling abnormality rather than directly repairing the gene.	mTOR inhibition in *TSC1/TSC2*; Ras–ERK modulation in *SYNGAP1*; receptor modulation in *GRIN* disorders	Leverages druggable enzymes/receptors; may cover multiple variants; small molecules may achieve broad CNS distribution	Does not repair causal gene; incomplete pathway normalization; chronic exposure; physiological importance of targeted pathways	Established clinical use–preclinical, depending on disorder
Engineered transcription-factor gene augmentation	AAV delivers a synthetic transcriptional regulator that binds endogenous regulatory DNA and increases expression of the intact gene without genome cleavage.	*SCN1A*—ETX101	Avoids delivery of a very large coding sequence; permits cell-type targeting; maintains endogenous protein	Durable vector expression; potential dose-dependent overactivation; sequence-specific regulator; immunogenicity/vector limitations	Early clinical development

**Table 2:** Methodology.

Component	Approach
Review design	Narrative review focused on precision, mechanism-informed, and potentially disease-modifying therapies for monogenic DEEs and NDD-E
Databases	PubMed/MEDLINE and ClinicalTrials.gov
Final search update	September 2026
Core disease terms	“developmental and epileptic encephalopathy,” “DEE,” “neurodevelopmental disorder,” “NDD,” “genetic epilepsy,” “monogenic epilepsy,” and individual disorder/syndrome names
Gene-specific searches	Individual causal gene names were searched separately with the corresponding disease phenotype and therapeutic terms
Precision/mechanistic terms	“precision medicine,” “precision therapy,” “targeted therapy,” “mechanism-based therapy,” “mechanism-informed therapy,” and “disease modification”
Gene/RNA therapeutic terms	“gene therapy,” “gene replacement,” “gene augmentation,” “antisense oligonucleotide,” “ASO,” “RNA therapy,” “RNA interference,” “siRNA,” “gene silencing,” “gene editing,” “CRISPR,” and “transcriptional activation”
Other therapeutic terms	“small molecule,” “pharmacological chaperone,” “protein stabilization,” “metabolic therapy,” “substrate supplementation,” “channel modulator,” and relevant pathway-specific terms
Preclinical evidence	Disease-relevant cellular, patient-derived cellular, organoid, and animal studies demonstrating therapeutic proof of concept
Human evidence	Case reports/series, expanded-access experience, prospective observational studies, and interventional clinical trials
ClinicalTrials.gov search	Individual genes and disorders combined with terms including “epilepsy,” “developmental encephalopathy,” “neurodevelopmental disorder,” “gene therapy,” “antisense oligonucleotide,” “RNA therapy,” “gene editing,” and “precision therapy”
Trial information extracted	Gene/disorder, therapeutic mechanism, intervention/product, phase, study population, ClinicalTrials.gov identifier, recruitment status, and available efficacy and safety findings
Primary inclusion principle	A defined mechanistic relationship between the intervention and the causal gene, functional consequence of the variant, or a well-established downstream disease pathway
Generally excluded	Conventional antiseizure medications and syndrome-directed symptomatic treatments without a specific gene-, variant-, or molecular-pathway rationale
Mechanism-informed therapy	Intervention that directly counteracts a defined molecular or functional abnormality associated with the causal gene or disease pathway
Potentially disease-modifying therapy	Intervention targeting the causal molecular defect or downstream disease biology with the potential to alter the broader disease trajectory beyond symptomatic seizure control
Evidence interpretation	Evidence was considered across the translational continuum from molecular/cellular proof of concept and animal models through individual human observations, early-phase studies, and controlled clinical trials

**Table 3:** Additional Mechanism-Informed Therapeutic Targets.

Gene(s)	Molecular Pathway/Disease Mechanism	Potential Mechanism-Informed or Disease-Modifying Therapies	Current Evidence/Translational Stage
*CDKL5*	X-linked serine/threonine kinase; neuronal maturation, dendritic growth, and synaptic maintenance; predominantly LoF.	Ganaxolone for seizure control; AAV-mediated CDKL5 replacement; secretable/cell-penetrating CDKL5 protein; splice-correcting ASOs, mRNA replacement, CRISPR correction, and X-chromosome reactivation.	Ganaxolone: established/approved clinical use (controlled phase 3 evidence); AAV replacement: preclinical; protein replacement: preclinical; ASO/mRNA/CRISPR/X-reactivation: preclinical/conceptual
*PCDH19*	Calcium-dependent cell adhesion; cellular-interference mechanism in heterozygous females and mosaic males; altered neurosteroid biology.	Ganaxolone based on reduced allopregnanolone/neurosteroid signaling; future gene-directed approaches.	Ganaxolone: controlled clinical trial (phase 2; efficacy endpoint not statistically significant); gene-directed approaches: preclinical
*TBC1D24*	Presynaptic membrane trafficking, synaptic-vesicle recycling, vesicular acidification, and oxidative-stress response; usually biallelic LoF/hypomorphic.	N-acetylcysteine amide or α-tocopherol; modulation of phosphoinositide/synaptojanin signaling; conceptual AAV, mRNA, CRISPR, or splice-modifying strategies.	Antioxidant approaches: preclinical; AAV/mRNA/CRISPR/splice modification: conceptual
*IQSEC2*	Postsynaptic ARF6 guanine-nucleotide exchange factor linked to NMDA-receptor signaling, AMPA-receptor trafficking, dendritic-spine development, and synaptic plasticity; pathogenic variants may cause LoF or abnormal/constitutive GEF activation.	Variant-matched ARF6/AMPA-pathway modulation; AMPAR potentiation; AAV-mediated *IQSEC2* replacement for LoF/null variants; mutant-allele suppression plus replacement for activating variants; CRISPR-based correction.	AMPAR modulation: preclinical; AAV replacement: preclinical; knockdown-and-replacement/CRISPR for activating variants: conceptual
*CNTNAP2*	CASPR2 is a neuronal cell-adhesion/neurexin-family protein involved in neuronal development, synaptic organization, Kv1-channel clustering, and excitation–inhibition balance; severe disease is particularly associated with biallelic LoF variants.	Oxytocin and Akt–mTOR inhibition; viral delivery of the CNTNAP2 intracellular domain (CICD); AAV-mediated MEIS2 expression; conceptual full-length *CNTNAP2* or mRNA replacement and CRISPR correction.	Oxytocin/Akt–mTOR modulation, CICD delivery, AAV-MEIS2: preclinical; full-length gene/mRNA replacement and CRISPR: conceptual
*NRXN1/NRXN2*	Presynaptic neurexins organize synapse formation and neurotransmitter release through multiple postsynaptic partners; effects are isoform- and context-dependent.	No established neurexin-specific therapy; gene restoration is complicated by large coding sequences, multiple promoters, extensive alternative splicing, and cell-type-specific isoforms.	Gene restoration: conceptual
*TANC2*	Postsynaptic scaffold interacting with PSD-95; regulates synaptic development and mTORC1/2 signaling; haploinsufficiency.	mTOR attenuation; ketogenic dietary therapy; gene augmentation.	mTOR modulation: preclinical; ketogenic therapy: case-level/anecdotal clinical evidence; gene augmentation: conceptual
*ADAM22*	Postsynaptic LGI1–ADAM22/23 synaptic complex; biallelic inactivation causes severe DEE.	Gene augmentation is conceptually attractive; no established ADAM22-specific pharmacologic or gene-directed therapy.	Gene augmentation: conceptual
*DEPDC5*	Component of the GATOR1 complex with NPRL2 and NPRL3; negatively regulates amino-acid-sensing mTORC1 signaling; LoF causes inappropriate mTORC1 activation.	Everolimus/rapamycin pathway inhibition; epilepsy surgery for focal second-hit disease; future AAV augmentation, CRISPR activation/correction, and downstream pathway targeting.	Everolimus/rapamycin: case-level/early clinical evidence; gene augmentation/CRISPR: preclinical/conceptual
*NPRL2/NPRL3*	GATOR1 complex; LoF causes persistent Rag-dependent mTORC1 activation and focal epilepsy/FCD spectrum.	mTOR inhibition with everolimus; gene augmentation/editing.	Everolimus: case-level/early clinical evidence; gene augmentation/editing: preclinical
*PIK3CA*	PI3K–AKT–mTOR signaling; postzygotic activating variants cause PROS and, when involving neural progenitors, mosaic brain malformations including megalencephaly, hemimegalencephaly, and FCD.	PI3Kα inhibition with alpelisib; mosaic-variant-selective silencing or gene editing.	Alpelisib: established/approved for severe systemic PROS; neurologic/epilepsy efficacy remains under clinical evaluation; variant-selective silencing/editing: conceptual
*ARX*	Homeobox transcription factor controlling forebrain patterning, interneuron development, and transcriptional programs; mechanism varies by variant class.	No established *ARX*-specific therapy; gene replacement, ASO, or genome-editing approaches.	Gene replacement/ASO/genome editing: conceptual
*CHD2*	ATP-dependent chromatin remodeling; haploinsufficiency disrupts transcriptional regulation and neuronal development.	Acetazolamide for photosensitive epilepsy; RNA-guided translational activation of the remaining *CHD2* allele; CHASERR/*CHD2* modulation; potential CRISPR-based transcriptional activation.	Acetazolamide: case-series clinical evidence; RNA-guided translational activation/CHASERR modulation: preclinical; AAV/CRISPR activation: conceptual
*EEF1A2*	Neuronal/muscle translation elongation factor with cytoskeletal and stress-response functions; variant-specific GoF, LoF, or dominant-negative effects.	Potential augmentation for true LoF variants or allele-selective suppression with replacement for toxic GoF/dominant-negative variants; requires variant-level functional characterization.	Variant-specific augmentation or suppression/replacement: conceptual
*PURA*	DNA/RNA-binding protein involved in transcriptional regulation, dendritic mRNA transport/local translation, and neuronal maturation; haploinsufficiency predominates.	Restoration of Pur-α expression; targeted poly(A)-tail mimetics (“mRNA boosters”); AAV gene augmentation and endogenous-gene activation.	mRNA boosters: preclinical; AAV augmentation/endogenous-gene activation: conceptual
*GRIA1–GRIA4*	AMPA-receptor subunits mediating fast excitatory neurotransmission; pathogenic variants cause variant-specific GoF or LoF through altered gating, desensitization, agonist sensitivity, trafficking, or ion permeability.	Perampanel for functionally confirmed GoF variants; AMPAR positive allosteric modulation for selected LoF variants; allele-selective ASO suppression; AAV augmentation, CRISPR-based activation/inactivation, and precise gene correction.	Perampanel for selected GoF variants: case-level clinical evidence; allele-selective ASO: preclinical; AAV/CRISPR/gene correction: conceptual

**Table 4:** Precision Therapeutics for Cell Metabolism, Protein Biosynthesis, and Degradation in DEEs.

Gene/Disease	Molecular Pathway and Disease Mechanism	Precision Therapeutics
*ALDH7A1*—pyridoxine-dependent epilepsy	Antiquitin deficiency disrupts lysine catabolism, causing accumulation of α-AASA/P6C, which inactivates pyridoxal-5′-phosphate (PLP) and disrupts neurotransmitter synthesis.	Pyridoxine; lysine-restricted diet and arginine supplementation reduce neurotoxic lysine metabolites. Early combined lysine-reduction therapy may improve developmental outcomes beyond pyridoxine alone.
*AMT*, *GLDC*—nonketotic hyperglycinemia	Deficiency of the glycine-cleavage system causes accumulation of glycine in plasma and CNS, producing excessive NMDA-receptor activation and severe neonatal encephalopathy.	Sodium benzoate lowers glycine; NMDA-receptor antagonism is mechanism-informed. Avoid valproate because it can impair glycine metabolism.
*ATP7A*—Menkes disease	Impaired ATP7A-mediated copper transport produces systemic and CNS copper deficiency with reduced activity of copper-dependent enzymes essential for neuronal function.	Early parenteral copper replacement, particularly copper histidinate, can partially bypass defective intestinal copper transport; benefit is greatest when initiated very early and depends on residual ATP7A function.
*BTD*—biotinidase deficiency	Failure to recycle biotin causes functional deficiency of multiple biotin-dependent carboxylases, producing organic acidemia, energy failure, and seizures.	Lifelong biotin supplementation directly bypasses defective biotin recycling.
*CAD*—CAD deficiency/DEE50	Biallelic *CAD* deficiency disrupts de novo pyrimidine synthesis, causing deficient uridine nucleotides with epilepsy, developmental impairment, and dyserythropoietic anemia.	Oral uridine/uridine monophosphate provides a pyrimidine salvage pathway that bypasses the metabolic block and can produce rapid seizure control and hematologic and developmental improvement.
*FOLR1*, *MTHFR*, *DHFR*, *SLC46A1/PCFT*—cerebral folate disorders	Defects in folate transport or metabolism reduce CNS availability of biologically active folates required for one-carbon metabolism, methylation, nucleotide synthesis, and neurotransmitter pathways.	Folinic acid for *FOLR1* and *DHFR*-related disease; folate/5-methyltetrahydrofolate and other pathway-specific cofactor replacement according to the molecular defect.
*GAMT*, *GATM*, *SLC6A8*—cerebral creatine deficiency syndromes	Defects in creatine synthesis (*GAMT*, *GATM*) or transport (*SLC6A8*) impair cerebral phosphocreatine-dependent energy buffering. *GAMT* deficiency additionally causes neurotoxic guanidinoacetate accumulation.	Creatine monohydrate for synthesis defects; *GAMT*: creatine plus arginine restriction and ornithine supplementation to reduce guanidinoacetate. Creatine alone has limited efficacy in *SLC6A8* deficiency; creatine with arginine/glycine has been explored.
Mitochondrial epilepsy genes—e.g., *FARS2*, *NARS2*, *RMND1*, *VARS2*, *PDSS2*, *COQ8A*, mtDNA genes	Defects in oxidative phosphorylation, mtDNA maintenance, mitochondrial translation, or CoQ10 biosynthesis impair ATP production and increase metabolic/oxidative stress, particularly in energy-dependent neurons.	Therapy should be genotype-specific: CoQ10 for primary CoQ10-biosynthesis defects; ketogenic therapy is particularly mechanism-based in pyruvate dehydrogenase deficiency and may be considered selectively in other mitochondrial epilepsies. Vatiquinone remains investigational and has not established efficacy broadly across mitochondrial epilepsies.
*MOCS1*—molybdenum cofactor deficiency type A	Failure of cyclic pyranopterin monophosphate synthesis prevents formation of molybdenum cofactor, causing sulfite oxidase deficiency, toxic sulfite accumulation, and rapidly progressive neonatal neurodegeneration.	Fosdenopterin (cPMP replacement) bypasses the *MOCS1*-dependent biosynthetic block and restores molybdenum-cofactor synthesis.
*OTC*—ornithine transcarbamylase deficiency	Urea-cycle blockade prevents efficient nitrogen disposal, causing hyperammonemia and secondary cerebral energy failure/neurotoxicity.	Protein restriction, nitrogen-scavenging therapy, citrulline/arginine supplementation as appropriate, and liver transplantation in selected severe disease; gene- and mRNA-based replacement strategies are under development. Avoid valproate.
*PHGDH*, *PSAT1*, *PSPH*—serine biosynthesis disorders	Impaired de novo L-serine synthesis reduces serine availability for protein, phospholipid, sphingolipid, and neurotransmitter metabolism during brain development.	L-serine supplementation, often with glycine depending on the defect; early or prenatal treatment may provide the greatest developmental benefit.
*PMM2*—PMM2-CDG	PMM2 deficiency reduces conversion of mannose-6-phosphate to mannose-1-phosphate, lowering GDP-mannose availability and causing defective N-linked protein glycosylation with multisystem and neurologic dysfunction.	Metabolic bypass: liposomal mannose-1-phosphate (GLM101; phase 2, NCT05549219). Pathway modulation: epalrestat increases residual PMM2 activity and improves glycosylation, although its phase 3 program was terminated for futility. D-galactose and acetazolamide have also been explored for selected manifestations. Gene-directed: AAV9-*PMM2* replacement improved glycosylation in patient cells and prevented or halted neurologic phenotypes in a mouse model, supporting future gene-replacement therapy.
*PNPO*—PNPO deficiency	PNPO catalyzes formation of PLP, the active vitamin B6 cofactor required for multiple neurotransmitter-synthesis enzymes; deficiency therefore produces functional PLP depletion and neonatal/infantile epilepsy.	Pyridoxal-5′-phosphate directly bypasses the enzymatic defect; some variants may also respond to pyridoxine.
*PLPBP*—PLPBP deficiency	Loss of PLP-binding/homeostasis protein disrupts intracellular PLP availability and vitamin-B6-dependent neurotransmitter metabolism.	Pyridoxine or pyridoxal-5′-phosphate, individualized according to biochemical and clinical response.
*POLG*—POLG-related mitochondrial disease	POLG is the catalytic polymerase responsible for mitochondrial DNA replication and repair. Pathogenic variants cause mtDNA depletion/deletions and progressive mitochondrial dysfunction, producing a spectrum that includes Alpers-Huttenlocher syndrome with developmental regression and severe epilepsy.	Deoxycytidine/deoxythymidine (dC/dT) supplementation aims to increase nucleotide substrate availability for mtDNA replication and has shown encouraging clinical and biomarker effects in an open-label phase 2 study (NCT04802707). PX578, a brain-penetrant small-molecule POLG activator developed by Pretzel Therapeutics, directly enhances POLG activity and is in preclinical development. Valproate should be avoided in *POLG*-related disease because of the risk of severe hepatotoxicity.
*SLC2A1*—GLUT1 deficiency syndrome	Impaired GLUT1-mediated glucose transport across the blood–brain barrier produces cerebral energy deficiency despite normal systemic glucose availability.	Ketogenic diet provides ketone bodies as an alternative cerebral fuel and is a prototypic metabolism-based precision therapy.
*SLC19A3*—biotin-thiamine-responsive basal ganglia disease	Defective thiamine transporter 2 reduces intracellular thiamine availability and thiamine-dependent energy metabolism, particularly during metabolic stress.	Early high-dose thiamine plus biotin can reverse or prevent neurologic deterioration and should be initiated promptly when suspected.
*SLC13A5*—citrate transporter disorder	Loss-of-function variants impair the neuronal sodium-dependent citrate transporter NaCT, disrupting citrate transport and cerebral metabolic homeostasis and causing neonatal/early-infantile epilepsy with developmental impairment.	Citrate supplementation and ketogenic therapy have been explored clinically with limited evidence. AAV9-*SLC13A5* gene replacement is in preclinical development; CSF delivery in *Slc13a5*-deficient mice reduced extracellular citrate, normalized electrophysiologic and sleep abnormalities, and rescued seizure susceptibility and mortality.

## Data Availability

No new data were created or analyzed in this study. Data sharing is not applicable to this article.

## Abbreviations

α-AASA	α-aminoadipic semialdehyde;
AAV	adeno-associated virus;
ATP	adenosine triphosphate;
CDG	congenital disorder of glycosylation;
CNS	central nervous system;
CoQ10	coenzyme Q10;
cPMP	cyclic pyranopterin monophosphate;
CSF	cerebrospinal fluid;
DEE	developmental and epileptic encephalopathy;
GDP	guanosine diphosphate;
GLUT1	glucose transporter type 1;
mRNA	messenger RNA;
mtDNA	mitochondrial DNA;
NaCT	sodium-dependent citrate transporter;
NMDA	N-methyl-D-aspartate;
P6C	Δ1-piperideine-6-carboxylate;
PLP	pyridoxal-5′-phosphate;
PMM2-CDG	phosphomannomutase 2 deficiency–congenital disorder of glycosylation.
